# Social support policy alternatives for older one-child parents in China: based on a decomposition and comparative analysis of subjective well-being

**DOI:** 10.3389/fpubh.2026.1723802

**Published:** 2026-02-23

**Authors:** Shengke Liu, Qiyan Zeng, Xiyu Miao, Yinchu Zeng

**Affiliations:** 1 School of Agricultural Economics and Rural Development, Renmin University of China, Beijing, China; 2 College of Economics and Management, Zhejiang A&F University, Hangzhou, Zhejiang, China

**Keywords:** aging, family planning policy, older one-child parents, social support policy alternatives, subjective well-being

## Abstract

**Background:**

Older parents from China’s 150 million one-child families have experienced a decline in subjective well-being. However, the relative importance of financial and time support via family versus public channels in association with life satisfaction remains unclear, posing a challenge for policy guidance.

**Objectives:**

This study aimed to evaluate and compare various social support policy alternatives for older one-child parents to identify potential measures that improve subjective well-being.

**Methods:**

Data were derived from four waves of the China Longitudinal Ageing Social Survey (2014–2020), comprising 7,517 observations from 4,170 parents aged 60 and above. A random-effects generalized ordered probit (REGOP) model was employed to analyze life satisfaction, while the Shapley decomposition method quantified the relative contribution of different support factors to inequality in predicted values. Group regression models and Fisher’s tests were employed to examine heterogeneity across living arrangements and urban–rural settings.

**Results:**

Family financial (*β* = 0.056/0.035, *p* < 0.01), public time (*β* = 0.202/0.340, *p* < 0.01), and family time (*β* = 0.038/0.034, *p* < 0.01) supports were significantly and positively associated with higher probabilities of being “Satisfied” and “Very satisfied,” whereas public financial support (*β* = 0.069, *p* < 0.01) was positively associated only with the “Satisfied” category. In terms of relative importance, financial support (11.94%) contributed more to subjective well-being inequality than time support (4.85%), and family support (12.01%) contributed more than public support (4.03%). Heterogeneity analyses revealed that financial and family time supports were significant and positive only in urban areas. Regarding living arrangements, the significant positive association of family time support was identified only in the solitary subgroup.

**Conclusion:**

For East Asian countries like China, prioritizing financial support and strengthening family function for prevalent one-child families might be potential strategies to address dual challenges of low fertility and aging. Furthermore, support policies could also be differentiated according to urban–rural contexts and living arrangements.

## 1 Introduction

Global population aging, driven by sub-replacement fertility and increasing longevity, has emerged as a defining demographic challenge of the 21st century. To safeguard the subjective well-being of older adults, international frameworks—such as the OECD’s long-term care strategies and the WHO’s “Healthy Ageing” initiative—have increasingly advocated for comprehensive social support systems that synergize state-provided public welfare with familial intergenerational solidarity. In promoting healthy aging, these frameworks posit a critical dynamic: while public transfers and social services are designed to alleviate the caregiving burden on adult children, informal family support, encompassing both financial transfers and time-based support, remains a cornerstone of older adults’ quality of life (1).

However, applying global frameworks to the societies that face more serious low fertility challenges and distinct old-age support institutions reveals profound tensions. In China, the prevalence of only-child families, a legacy of the coercive one-child policy between 1979 and 2015 (2), has engendered a “shrinking support base,” placing the weight of old-age support on a sole adult child. In spite of having established a basic pension system, compared with developed Western countries, China relies more heavily on family members as the main provider of old-age support. Aiming to support 150 million older one-child parents (3), who have experienced a decline in subjective well-being compared with their counterparts (4), many initiatives (please refer to Table A1) have also been proposed in the context of the Chinese traditional family-based support model. However, policymakers currently confront several dilemmas in designing old-age support systems. First, regarding resource allocation, how should they prioritize between financial (subsidies) support and time (older adults care services) support? Second, should government intervention aim to bolster family caregiving capacity—for instance, by providing subsidies to one-child families and promoting flexible work arrangements—or should it seek to mitigate the family’s role by expanding third-party service provision and channeling pension benefits directly to older adults? Furthermore, the applicability of these support policies across diverse contexts, such as rural–urban settings and different living arrangements, also requires thorough investigation.

This study investigates social support policy alternatives for older one-child parents. Specifically, first, with finite old-age resources, what is the relative contribution of financial and time support factors to subjective well-being inequality among older one-child parents? Second, in the Chinese context, what is the primary source of subjective well-being inequality between family and public provision channels? Third, given the diverse socio-cultural and old-age security systems between rural and urban areas in China, as well as the increasing prevalence of solitary living among one-child families (5, 6), are the associations between various social supports and subjective well-being heterogeneous? By answering these three questions, this study will provide new insights for balancing the social adequacy of support systems with financial sustainability, and it could also help in rethinking the capacity of families to support older members from one-child families.

## 2 Literature review

### 2.1 Theoretical framework

Social support is early defined as information leading one to believe that he or she is cared for, loved, esteemed, and a member of a network of mutual obligations (7). Support systems are commonly dichotomized into formal and informal types based on the source of assistance. Formal support denotes institutional aid from state and social organizations, in contrast to informal support, which encompasses resources stemming from personal networks, including family, kin, and neighbors (8). In terms of support properties, House (1981) classified social support into four distinct types: instrumental support, emotional support, informational support, and appraisal support (9). Several studies examining upward intergenerational transfers have further classified old-age support into two distinct categories: time-based and financial support (10, 11). Time-based support refers to caregiving and emotional assistance provided by adult children to their older parents, whereas financial support comprises monetary transfers and in-kind gifts given to older adults (12, 13). The complexity in classifying social support arises from its inherent multi-level interactions and the varied research perspectives adopted by scholars. In this study, we categorized social support resources into two primary types—time-based support and financial support—originating from family and public channels, respectively.

The Psychological Immunity Model, proposed by Cobb (7) in 1976, suggested that social support buffered the negative effect of stress on mental health through multiple pathways encompassing cognitive reappraisal, behavioral activation, and physiological buffering. Regarding the behavioral activation mechanism, social support networks promote adaptive coping behaviors through resource supply (e.g., financial assistance or information sharing). Furthermore, the main effect model, proposed by Thoits (14), argued that social support directly increases individual happiness, regardless of stress. Despite subtle divergences in the underlying mechanisms across theoretical frameworks, a broad consensus exists regarding the substantial and multifaceted impact of social support on mental health.

### 2.2 The associations of different types of social support with subjective well-being

As individuals age, older adults’ need for various forms of old-age assistance and resources intensifies, making social support increasingly crucial in evaluating their subjective well-being. Despite cultural differences between Eastern and Western societies, previous studies that aggregated financial and time-based support have consistently demonstrated a positive association between social support and life satisfaction among older adults (15–18). Moving beyond this aggregate level of analysis, regarding financial support, studies conducted in Nigeria and China—utilizing methodologies such as qualitative approaches and structural equation modeling—have demonstrated that state-sponsored pensions and material support from family or community networks are vital for enhancing older adults’ life satisfaction (19, 20). Likewise, with respect to time-based support, research from the US, Turkey, and Japan employing quantitative regression models indicates that social support, encompassing instrumental, emotional, and informational assistance, is positively associated with older adults’ life satisfaction (21–23). While this research has demonstrated that both financial and time-based support are positively associated with older adults’ subjective well-being, the two forms of support have typically been examined in isolation rather than within a unified analytical framework. Building on these studies, Chinese scholars have compared the relative importance of various social support types for older adults’ subjective well-being; however, the findings remain inconsistent. Using a structural equation model, one study by Li et al. (24) found that the influence of time-based support on subjective well-being is more pronounced than that of financial support in older adult patients with chronic diseases, while another study using an OLS regression model by Zhan et al. (25) indicated that intergenerational financial support has the most pronounced effect, followed by emotional and instrumental support, on the subjective well-being of general older adults. The discrepancies in conclusions may stem from the heterogeneity of older adult subgroups, diverse operationalizations of social support, and varying analytical approaches across studies.

### 2.3 The family function in old-age support for one-child family

Although the relationship between formal and informal older adults care services for general older families has been extensively debated, the literature on social support for one-child families is scarce. Additionally, in the face of increasing old-age budget pressure, Western nations and China are at divergent stages concerning family function. Western governments are inclined to revert from costly formal care to family-based older adults care services aiming to curtail public expenditures (26). Some European studies revealed that family informal care substituted for low-skill formal services but complemented professional healthcare and outpatient services (26, 27). A Canadian study suggested a substitution relationship between informal care and formal home care in older individuals (28). Despite the existence of regional variation, there is a consensus that informal and formal care increasingly collaborate as older adults’ needs intensify. Conversely, in China, family-based older adults care services is entrenched as the primary mode, with public older adults care services serving as a supplementary rather than a substitute option. Research indicated that formal care did not substitute but rather supplemented family care (29), and the Social Insurance of the Old-age Pension for Urban and Rural Residents (SIOPURR) in China had no significant impact on upward intergenerational transfers from adult children (30). Other studies suggested that formal care had both complementary and substitution effects, with an overall positive impact on informal care (31). Given the distinct structure of one-child families and the divergent modernization trajectories between Western and Chinese aging societies, the evolving role of the family—whether it should be reinforced or weakened—deserves further examination. This understanding is essential for Chinese policymakers to prioritize intervention channels aimed at supporting older one-child parents who have experienced a decline in subjective well-being.

### 2.4 The living arrangement and urban–rural differences of older family

The living arrangements of one-child families and urban–rural disparities are also pivotal when crafting old-age social support policies. Although China and South Korea are both East Asian countries belonging to the Confucian cultural sphere, trends in family living arrangements have diverged between the two nations recently. A majority of Korean young adults live with their parents, while Chinese young adults have experienced large shifts to solitary living in their living arrangements (32). Surveys indicate that approximately half of China’s older adults prefer to live separately from their adult children (33), a choice more prevalent among those with better health (34, 35), higher socio-economic status (36), and higher income (37). In some East Asian countries, proximity influences the type of support that adult children provide, with those living closer offering time support (emotional comfort and visits), while distant children provide financial aid (38, 39). This dynamic complicates the policy environment. In contrast to the comprehensive welfare systems found in OECD nations, China’s three-tier pension system is embedded in an urban–rural dual institutional framework. Consequently, there are significant disparities in benefit levels, growth rates, and government support, with the Old-age Insurance System for Government Agencies and Institutions (OISGAI) and Basic Old-age Insurance System for Urban Employees (BOISUE) surpassing the SIOPURR (40). These disparities may account for the mixed results observed in previous studies, highlighting the critical need to account for living arrangements and urban–rural heterogeneity when analyzing support for older one-child parents.

### 2.5 Objectives

While prior studies have advanced our understanding of how social support influences subjective well-being among older one-child parents, the existing literature has not yet fully elucidated the complexities underlying this relationship, leaving several questions unresolved. First, although time-based and financial social support are recognized as fundamental, there is a dearth of quantitative research on prioritizing government intervention in old-age resource allocation, especially for one-child families. Second, the debate on whether to strengthen or weaken family functions through government support for one-child families remains unresolved, with a current focus on the mutual relationship between general formal and informal old-age care. Third, urban–rural disparities and solitary living patterns, which might affect older adults’ needs and policy efficacy, have been overlooked in discussions of old-age social support.

This study steps forward by applying the Shapley value decomposition method to identify the relative contribution shares of two old-age resources (time-based and financial support) and two provision channels (family and public) to the one-child parents’ subjective well-being inequality, which responds to the question of policy priority and orientation. Furthermore, by examining the relationships of old-age social support and subjective well-being within urban–rural and various living arrangement subgroups, we evaluate the implementation conditions of the support policies.

## 3 Materials and methods

### 3.1 Data source

This study utilized secondary data from the China Longitudinal Aging Social Survey (CLASS), a comprehensive and nationally representative longitudinal survey of individuals aged 60 or above, conducted by Renmin University of China. The data sources and design reports are available at http://class.ruc.edu.cn. This survey was conducted in 2014, 2016, 2018, and 2020, with 11,511, 11,471, 11,417, and 11,398 individuals being interviewed in each round, respectively. It covered 28 provinces, including municipalities and autonomous regions, and extends to 134 counties and 462 villages or neighborhood communities across mainland China. The CLASS was approved by the Academic Committee of the Institute of Gerontology at Renmin University of China and provided rich demographic, health, socio-economic, family, and community characteristics information for our research. A stratified probability sampling method was employed, with counties as primary units, villages/neighborhoods as secondary units, and older adults aged 60 or above as respondents. The trained interviewers collected the data through face-to-face interviews. After introducing the research purposes to respondents, the interviewers documented detailed information on the process of obtaining informed consent, which was stored by the Institute of Gerontology and the National Survey Research Center at Renmin University of China.

**Figure 1:**
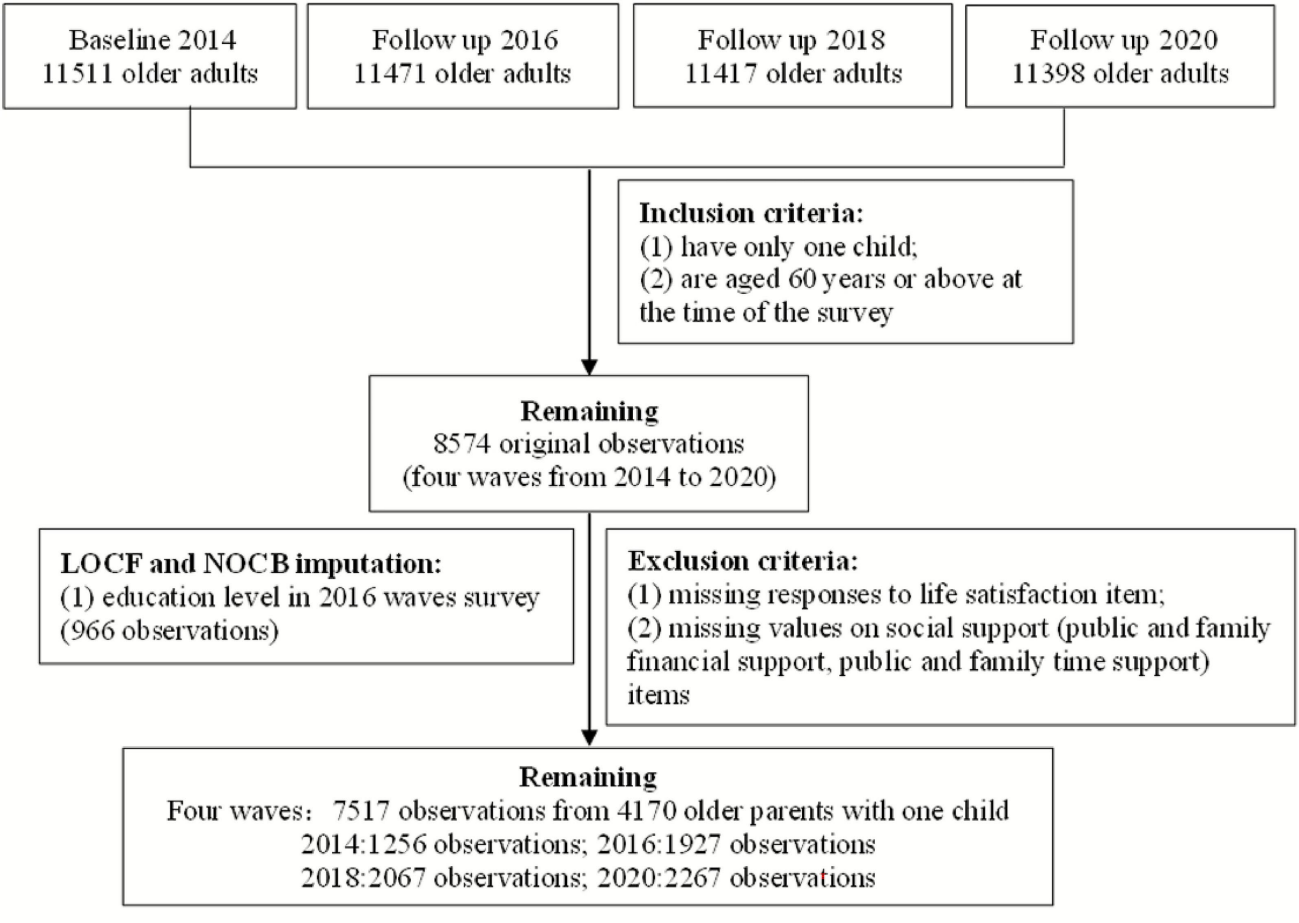
Sample selection flow diagram.

Focusing on older parents from one-child families, we limited the sample to respondents with a single child who were aged 60 or older. This resulted in an initial sample of 8,574 observations across four waves of CLASS between 2014 and 2020. We then excluded cases with missing data on the dependent variable (life satisfaction) or key independent variables (social support metrics covering financial and time-based support from both public and family sources). Finally, for the 358 system-missing observations regarding the control variable of education level in 2016, we applied Last Observation Carried Forward (LOCF) and Next Observation Carried Backward (NOCB), considering “education” to be a time-invariant characteristic. After addressing random missingness in key variables through listwise deletion, LOCF, and NOCB imputation, the final analytical sample yielded 7,517 observations from 4,170 older one-child parents (see Figure 1). The data employed in this paper constitute an unbalanced panel: Regarding the 7,517 observations, older one-child parents were surveyed across varying waves, with 28.6% participating in one wave, 22.8% in two waves, 39.7% in three waves, and 8.9% in four waves.

### 3.2 Variables and measurements

Dependent variable. For the measurement of older one-child parents’ subjective well-being, this study used life satisfaction as a proxy that represents older individuals’ perceptions of their current living conditions and quality of later life (41). Many previous studies highlighted the importance of life satisfaction to the subjective well-being levels among adults (42). In the survey, older respondents rated their overall life satisfaction on a five-point scale, as shown in Table 1. We then recoded it from 1 (Very dissatisfied) to 5 (Very satisfied), with higher scores indicating better subjective well-being.

**Table 1:** Questionnaire items for dependent variables and independent variables.

Category	Variables	Questions	Options
Dependent variable	Life satisfaction	Overall, are you satisfied with your current life?	(1) Very satisfied, (2) Satisfied, (3) Neutral, (4) Dissatisfied, (5) Very dissatisfied
Independent variable	Family financial support	In the past 12 months, has this child given you (or your surviving spouse living with you) money, food, or gifts? If yes, what was the total value of these provisions?	(1) Never, (2) 1–199 RMB, (3) 200–499 RMB, (4) 500–999 RMB, (5) 1,000–1,999 RMB, (6) 2,000–3,999 RMB, (7) 4,000–6,999 RMB, (8) 7,000–11,999 RMB, (9) 12,000 RMB and above
	Public financial support	How much is your monthly Social Pension for Urban and Rural Residents (combining the former Urban Resident Social Pension and New Rural Social Pension)?	Actual pension benefit received (unit: thousand RMB/month)
	Family time support	In the past 12 months, how often did this child help you with household chores? In the past 12 months, how often did you meet with this child? In the past 12 months, how often did you contact this child (via phone, WeChat, or other communication methods)?	(1) Almost every day, (2) once a week at least, (3) once a month at least, (4) several times a year, (5) almost never
	Public time support	Have you used any of the following services provided by your community?	(1) Home visit, (2) medical escort service, (3) day care center for the older adult, (4) psychological counseling

Independent variables. Social support for older one-child parents was measured along two dimensions: the nature of support (financial vs. time) and the provision channel (family vs. public), resulting in four types of social support: family financial, public financial, family time, and public time support. Specifically, we measured family financial support by the total financial support provided by their child, while public financial support was measured by the amount of SIOPURR received from the government. The family financial support, including money, food, and gifts, in the past year, had nine categories as shown in Table 1, and we recoded it into a continuous variable as 0–8 in order. As for the public financial support, we used the item of SIOPURR received per month in the questionnaire as shown in Table 1, and converted its unit to thousand RMB. Family time support referred to the time investment by adult children in providing care and fostering emotional bonds with their older adult parents. In this study, it was quantified using a three-item instrument (see Table 1). Each item was rated based on frequency, with five options that were ordinally coded from 1 to 5. A total score was then computed by summing these values, forming a continuous variable where higher scores indicated more frequent family time support. Public time support referred to the time investment by public organizations in offering care and fostering emotional bonds with older adult parents. In this study, it was operationalized as the total number of community older adults care services utilized by older one-child parents, assessed via a single item with four options (see Table 1). A higher sum score indicated a greater level of such support.

Control variables. With reference to the relevant theories and previous studies (37, 43, 44), we controlled for characteristic variables that were correlated to SWB (see Table 2). Specifically, demographic characteristics included gender, age, education level, and marital status. Physical health characteristics included ADL, IADL, and chronic diseases. Among these variables, ADL refers to the activities of daily living scale for older adults with 10 items (e.g., Feeding, Dressing, and Toileting), and IADL refers to the instrumental ability of daily living scale for older adults, which consists of eight items (e.g., Transportation, Housekeeping, Managing Finances, and Shopping). Socio-economic characteristics included primary means of livelihood, house asset, household consumption, OISGAI or BOISUE receiving, social participation, and social network. Among these variables, the means of livelihood refers to a range of resources by which the older adult sustain their lives, including nine items (e.g., pension, financial support from children, and rent), and these sources were divided into three categories: leave/retirement/pension or child support, income from self-employment or employment of a spouse, and income from other sources. House asset refers to the amount of older parents’ homeownership. Household consumption refers to the total consumption of older parents’ family per month. OISGAI or BOISUE receiving indicates whether older parents received public sector or enterprise pensions. Social participation indicates whether older parents participate in community volunteer work (e.g., neighborhood watch patrols and mediating neighborhood disputes). Social network refers to the number of friends with whom older parents maintain regular contact, share confidences, and seek help. Family characteristics included the gender of children, marital status of children, and living arrangement. Community characteristics included urban–rural attribute. In addition, the time effect and regional effect were also controlled. The measurement and coding of these controlled variables are shown in Table 2.

**Table 2:** Summary statistics of control variables and their relationship with life satisfaction (*N* = 4,170).

Category	Variable	Variable type	*n* (%)/Mean (S.D.)	Corr. Coeff.
Demographic characteristics	Age	Integer	66.437 (6.268)	−0.027*
	Gender	Binary		0.000
		0: Female	196 (47.19%)	
		1: Male	2,202 (52.81)	
	Education level	Integer		0.075***
		0: No school	463(11.10%)	
		1: Old-style private school or literacy class	92(2.21%)	
		2: Elementary school	990 (23.74%)	
		3: Middle school	1,492 (35.78%)	
		4: High school	753 (18.06%)	
		5: Bachelor’s degree or more	380 (9.11%)	
	Marital status	Binary		0.095***
		0: Single, divorced, or widowed	881 (21.13%)	
		1: Married	3,289 (78.87%)	
Physical health characteristics	ADL	Integer	17.737 (1.336)	0.125***
	IADL	Integer	13.391 (1.86)	0.133***
	Chronic diseases	Binary		−0.105***
		0: No	155 (37.36%)	
		1: Yes	261 (62.62%)	
Socio-economic characteristics	Primary means of livelihood	Integer		−0.086***
		0: Leaving/retirement pension or children financial support	3,050 (73.14%)	
		1: Self-employment or employment of spouse	675 (16.19%)	
		2: Other sources of income	445 (10.67%)	
	House asset	Integer		0.070***
		0: 0	173 (4.15%)	
		1: 1	3,546 (85.04%)	
		2: 2	411 (9.86%)	
		3: 3 or above	40 (0.96%)	
	Household consumption	Real	1.247 (1.165)	−0.005
	OISGAI or BOISUE receiving	Binary		0.062***
		0: No receiving	1704 (40.86%)	
		1: Receiving	2,466 (59.14%)	
	Social participation	Binary		0.042***
		0: No participation	3,395 (81.41%)	
		1: Participation	775 (18.59%)	
	Social network	Real	2.194 (1.164)	0.093***
Family characteristics	Gender of children	Binary		−0.029*
		0: Female	1,477 (35.42%)	
		1: Male	2,693 (64.58%)	
	Marital status of children	Binary		0.045***
		0: Single, divorce, or widowed	595 (14.27%)	
		1: Married	3,575 (85.73%)	
	Living arrangement	Binary		0.058***
		0: Solitary living (single or with spouse)	2,409 (57.77%)	
		1: Co-residing living (with children)	1761 (42.23%)	
Community characteristics	Urban–rural attribute	Binary		0.055***
		0: Rural area	1,057 (25.35%)	
		1: Urban area	3,113 (74.65%)	

The table presents the variable characteristics for the sample of surveyed households at baseline (first observation), tracking records are not duplicated. *p < 0.10, ***p < 0.01. Unit of household consumption is “thousand RMB.” Corr. Coeff.: correlation coefficient.

### 3.3 Statistical analysis

The Random-Effects Generalized Ordered Probit (REGOP) model. Given the moderate left skewness (skewness = −0.74) of the ordered categorical variable “life satisfaction,” its original 5-point scale was first collapsed into three categories: “Dissatisfied” (1–3), “Satisfied” (4), and “Very satisfied” (5)—to mitigate variability arising from random effects (45, 46). To address the potential violation of the parallel regression assumption inherent in the standard Ordered Probit model, we employ the REGOP model. The REGOP model does not restrict the coefficients to be constant across outcome thresholds and allows the effects of explanatory variables to vary over different levels of life satisfaction (47). Furthermore, the model incorporates an individual-specific random term ( $u_{i}$ ) to account for unobserved heterogeneity in panel data. The model is estimated via maximum simulated likelihood, assuming normality for both the idiosyncratic error and the random effect. A likelihood ratio test (LR test) was conducted to compare the standard REOP model with the REGOP model. As shown in the bottom row of Table 3, the LR test result rejected the null hypothesis of the parallel regression assumption ( $\chi^{2}$ = 205.55, df = 32, p <0.001). This indicates that the coefficients of the explanatory variables vary across different categories of “life satisfaction.” Therefore, the REGOP model is preferred for estimation. The random-effect parameter rho (*ρ*) in the REGOP model was 0.468 and was found to be significant at the 1% level (see the bottom row in Table 3), indicating that 46.8% of the total variation is due to unobservable individual heterogeneity, which justifies the use of a random-effects specification.

**Table 3:** Results of the REGOP model of life satisfaction and robust test.

Variables	(1) REGOP: full sample		(2) REGOP: subgroup sample (aged 60–70)		(3) FEOLS: full sample
	Satisfied	Very satisfied	Satisfied	Very satisfied	Life satisfaction
Public financial support	0.069*** (0.016)	−0.006 (0.016)	0.079*** (0.019)	0.0001 (0.018)	0.017* (0.010)
Family financial support	0.056*** (0.010)	0.035*** (0.011)	0.059*** (0.011)	0.024** (0.011)	0.015* (0.009)
Public time support	0.202*** (0.061)	0.340*** (0.057)	0.181** (0.072)	0.329*** (0.067)	0.130** (0.054)
Family time support	0.038*** (0.010)	0.034*** (0.010)	0.032*** (0.010)	0.024** (0.011)	0.013*(0.008)
Age	0.008* (0.004)	−0.001 (0.005)	−0.022** (0.010)	−0.019* (0.010)	0.043**(0.019)
Marital status	0.247*** (0.063)	0.062 (0.068)	0.359*** (0.072)	0.119 (0.079)	0.062 (0.105)
Gender	−0.052 (0.050)	0.037 (0.051)	−0.030 (0.056)	0.046 (0.056)	—
Education level					
Literacy class	0.188 (0.166)	0.132 (0.166)	0.716*** (0.238)	0.310 (0.220)	—
Secondary school	−0.052 (0.083)	−0.210** (0.087)	0.003 (0.083)	−0.206* (0.109)	—
Junior high school	0.037 (0.088)	−0.181** (0.092)	0.034 (0.106)	−0.212* (0.111)	—
High school	0.057 (0.100)	−0.069 (0.103)	0.053 (0.119)	−0.066 (0.121)	—
College or above	0.328*** (0.126)	0.132 (0.121)	0.329** (0.145)	0.115 (0.139)	—
ADL	0.115*** (0.022)	0.098*** (0.026)	0.108*** (0.033)	0.143*** (0.041)	0.103*** (0.030)
IADL	0.054*** (0.017)	0.0004 (0.019)	0.059** (0.024)	0.001 (0.026)	0.039**(0.020)
Chronic diseases	−0.355*** (0.050)	−0.467*** (0.049)	−0.386*** (0.056)	−0.426*** (0.054)	−0.218***(0.035)
Marital status of children	0.170** (0.073)	−0.039 (0.076)	0.203** (0.079)	0.037 (0.082)	0.044 (0.065)
Gender of the children	−0.070 (0.052)	−0.052 (0.052)	−0.039 (0.057)	−0.092 (0.057)	—
Primary income from working^a^	−0.002 (0.071)	0.067 (0.069)	−0.037 (0.079)	0.028 (0.077)	−0.056 (0.048)
Primary income from others^a^	−0.191*** (0.074)	−0.221*** (0.081)	−0.199** (0.088)	−0.278*** (0.096)	−0.045 (0.050)
Living arrangement	0.160*** (0.050)	0.263*** (0.052)	0.171*** (0.057)	0.208*** (0.058)	0.156***(0.041)
Social participation	−0.016 (0.051)	0.064 (0.052)	−0.003 (0.058)	0.048 (0.057)	0.041 (0.033)
Social network	0.055*** (0.021)	0.087*** (0.021)	0.065*** (0.023)	0.086*** (0.024)	0.060***(0.018)
Urban–rural attributes	0.033 (0.072)	−0.005 (0.075)	0.049 (0.084)	−0.008 (0.088)	0.172 (0.136)
House asset	0.096* (0.055)	0.162*** (0.054)	0.140** (0.062)	0.190*** (0.059)	0.088** (0.040)
OISGAI or BOISUE receiving	0.163** (0.071)	−0.264*** (0.073)	0.209** (0.083)	−0.286*** (0.084)	0.047 (0.061)
Household consumption	−0.029** (0.013)	−0.029* (0.017)	−0.020 (0.019)	−0.021 (0.019)	−0.008 (0.011)
Region effect	Controlled	Controlled	Controlled	Controlled	—
Time effect	Controlled	Controlled	Controlled	Controlled	Controlled
*ρ* (std. err.)	0.468(0.019) (*p*-value <0.001)		0.438(0.024) (*p*-value < 0.001)		0.544
Parallel slope assumption LR test (REGOP vs. REOP)	205.55 (d.f. = 32) (*p*-value <0.001)		168.05 (d.f. = 32) (*p*-value < 0.001)		—
Sample size	7,517		5,728		5,365

*p < 0.10, **p < 0.05, ***p < 0.01. Standard errors are provided in parentheses. “a” indicates that the control group is older one-child parents whose primary means of livelihood is from “leaving/retirement pension” or “financial support from children.” *ρ* is the ratio of the unobserved individual heterogeneity variance to the total variance.

The REGOP model allows the coefficients of explanatory variables to vary across different categories of “life satisfaction.” The underlying latent variable, $LS_{\mathrm{it}}^{*}$ , is specified in Equation (1) as follows:


$$LS_{\mathrm{it}}^{*}=x_{\mathrm{it}}^{\prime }\beta_{j}+u_{i}+\in_{\mathrm{it}}$$ (1)

where $x_{\mathrm{it}}$ represents a vector of explanatory variables (four forms of social support and other control variables) for individual i at time t . Unlike the standard model, the coefficient vector $\beta_{j}$ is allowed to differ for each outcome category j (in this case, j = 3), thereby capturing the heterogeneous effects of the regressors. The term $u_{i}$ denotes the individual-specific random effect, accounting for time-invariant unobserved heterogeneity, while $\in_{\mathrm{it}}$ is the idiosyncratic error term. We assume that $u_{i}∼N(0,\sigma_{u}^{2})$ and $\in_{\mathrm{it}}∼N(0,1)$ .

The observed ordinal outcome, $LS_{\mathrm{it}}$ , is derived from the latent variable through the following threshold mechanism in Equation (2):


$$LS_{\mathrm{it}}=j\;\;\text{if and only if}\;\;\mu_{j-1}<LS_{\mathrm{it}}^{*}\leq \mu_{j}$$ (2)

where $\mu_{j}$ are the cut-points (thresholds) to be estimated, with $\mu_{0}=-\infty$ and $\mu_{J}=+\infty$ .

Conditional on the random effect $u_{i}$ , the probability that individual i chooses category j at time t is given by Equation (3):


$$P(LS_{\mathrm{it}}=j|u_{i})=\Phi (\mu_{j}-x_{\mathrm{it}}^{\prime }\beta_{j}-u_{i})-\Phi (\mu_{j-1}-x_{\mathrm{it}}^{\prime }\beta_{j-1}-u_{i})$$ (3)

where Φ(·) is the standard normal cumulative distribution function. To obtain the unconditional likelihood function, we integrate out the random effect $u_{i}$ by assuming it follows a normal distribution. The likelihood function involves integrating over the distribution of the random effect $u_{i}$ . Since this integral does not have a closed-form solution, the model is estimated via maximum simulated likelihood (MSL) using the user-written command regoprob2 (48) in Stata16.

Shapley decomposition. Life satisfaction was recoded into a binary variable. Specifically, responses of 4 and 5 were categorized as “Satisfied,” while responses of 1, 2, and 3 were categorized as “Dissatisfied.” Based on the results of the probit model, the Shapley decomposition method (49) was utilized to decompose the inequality of the probability of being “Satisfied.” This technique quantifies the relative contribution of different old-age social support factors from different channels to life satisfaction inequality, with higher contribution shares indicating potential priority and orientation of policy intervention. The method originates from Shapley (50) in cooperative game theory and was extended to inequality and regression-based decomposition by (51, 52). The Shapley method satisfies the standard axioms of efficiency, symmetry, and dummy player, and provides an additive decomposition in which the contributions of all factors sum exactly to the total change in the outcome of interest (51, 53). A key advantage over alternative decomposition techniques, such as the Oaxaca–Blinder decomposition or the Fields regression-based approach (54), is that the Shapley value does not depend on the order of variables and naturally accommodates nonlinear models. Given the nonlinear nature of our probit model, the Shapley decomposition is particularly well suited to our empirical setting. The Shapley approach has been widely used to decompose inequality of opportunity (55), life satisfaction (56), and income inequality (46) in recent studies, and we follow this established convention in our implementation.

Group regression model and Fisher’s test. A set of group regression models were employed to explore the heterogeneous effects of various old-age social support on life satisfaction across different living arrangements and urban–rural settings. Fisher’s test was used to examine whether the coefficient differences were significant between two subgroups, with significantly larger coefficient in one subgroup indicating the conditions under which the support policies were more effective. Furthermore, the use of multiple subgroup regression analyses and Fisher’s tests was applied to examine heterogeneity in the associations between various types of social support and the subjective well-being of older one-child parents. However, the multiple comparisons may lead to an inflated risk of Type I error. To address this, we applied the Benjamini–Hochberg (BH) procedure to control the false discovery rate (FDR) at 5%. After FDR correction, some heterogeneity in association (e.g., association difference of public financial support between urban and rural subgroups) remained statistically significant, whereas other heterogeneity in the association (e.g., association difference of public time support between urban and rural subgroups) no longer met the significance threshold. Our core conclusions rest on findings that remain robust to this adjustment.

## 4 Results

### 4.1 Descriptive statistics

Table 4 presents the descriptive statistics of the dependent and main independent variables. Regarding the dependent variable, as shown in Table 4, older one-child parents in urban areas and those living in solitary patterns had significantly higher life satisfaction levels compared with rural counterparts and those living in co-residing patterns, respectively. As for financial support among the main independent variables, older one-child parents in urban areas received significantly more family financial support and public financial support than rural ones. However, older one-child parents living in solitary patterns received significantly more family financial support but less public financial support than those living in co-residing patterns. Regarding time support, older one-child parents in urban areas received significantly more family time support but used less public time support than rural ones. Although older one-child parents living in co-residing patterns received significantly more family time support than those living in solitary patterns, no significant difference was found between these two subgroups.

**Table 4:** Summary statistics of the dependent and main independent variables (*N* = 4,170).

Statistic	Subgroup	Dependent variable	Independent variable			
		Life satisfaction	Family financial support	Public financial support	Family time support	Public time support
Mean (S.D.)	Full sample	3.956 (0.843)	3.681 (2.362)	0.347 (1.411)	10.424 (2.643)	0.076 (0.348)
Mean (S.D.)	Rural	3.876 (0.880)	3.031 (2.109)	0.264 (1.127)	9.963 (2.850)	0.112 (0.427)
	Urban	3.983 (0.830)	3.901 (2.404)	0.375 (1.500)	10.581 (2.551)	0.064 (0.315)
Diff. (S.E.)		−0.107*** (0.030)	−0.870*** (0.083)	−0.111** (0.050)	−0.618*** (0.094)	0.048*** (0.012)
95% CI		[−0.166, −0.048]	[−1.033, −0.707]	[−0.209, −0.012]	[−0.802, −0.434]	[0.024, 0.072]
Mean (S.D.)	Co-residing	3.900 (0.849)	3.567 (2.513)	0.410 (1.517)	11.072 (2.711)	0.068 (0.321)
	Solitary	4.000 (0.837)	3.764 (2.243)	0.301 (1.328)	9.951 (2.488)	0.081 (0.366)
Diff. (S.E.)		−0.099*** (0.026)	−0.197*** (0.074)	0.109** (0.044)	1.120*** (0.081)	−0.013 (0.011)
95% CI		[−0.151, −0.047]	[−0.342, −0.051]	[0.022, 0.196]	[0.961, 1.280]	[−0.035, 0.008]

The table presents the variable characteristics for the sample of interviewed households at baseline (first observation), tracking records are not duplicated. **p < 0.05, ***p < 0.01. S.D.: standard deviation; S.E.: standard error; Diff.: difference; CI: confidence intervals.

In addition, Table 5 presents the correlation matrix for the major variables. Four main independent variables, namely family financial support, public financial support, family time support, and public time support, are all reported to be positively associated with the life satisfaction of older one-child parents at the 1% significance level. With the exception of modest correlations between family financial support and both public financial support (0.098) and family time support (0.139), no other significant correlations were observed among the main independent variables. Furthermore, multicollinearity diagnostics in Table 6 show that all VIFs are below 3.77 (mean VIF = 1.56), indicating the absence of multicollinearity concerns.

**Table 5:** The correlation matrix for major variables (*N* = 4,170).

Variables	Life satisfaction	Family financial support	Public financial support	Family time support	Public time support
Life satisfaction	1.000	—	—	—	—
Family financial support	0.118***	1.000	—	—	—
Public financial support	0.059***	0.098***	1.000	—	—
Family time support	0.072***	0.139***	0.013	1.000	—
Public time support	0.058***	−0.009	−0.005	0.021	1.000

The table presents the variable characteristics for the sample of surveyed households at baseline (first observation), tracking records are not duplicated. ***p < 0.01.

**Table 6:** Variance inflation factors (VIFs) for independent variable and control variables (*N* = 4,170).

Statistic	Family financial support	Public financial support	Family time support	Public time support	Control variable
VIF	1.13	1.36	1.23	1.05	<=3.77
1/VIF	0.887	0.735	0.816	0.954	<=0.265
Mean VIF	1.56				

Among control variables, the maximal VIF and 1/VIF are 3.77 and 0.265, respectively.

Table 2 summarizes the survey respondents’ demographic, physical health, socio-economic, family, and community characteristics, as well as their relationships with life satisfaction. The average age of older one-child parents was 66.437 years, and among them, 52.81% were male. The educational level of the respondents predominantly ranged from elementary to high school, with middle school constituting the largest proportion at 35.78%. Additionally, 78.87% of older one-child parents were married. Regarding the physical health of older one-child parents, 62.62% reported having a chronic disease, and the mean values of ADL and IADL were 17.737 and 13.391, respectively. In terms of socio-economic characteristics, it was reported that 73.14% of older one-child parents relied on leave/retirement/pension or children’s support as their primary means of livelihood. Homeownership was prevalent among older one-child parents, at a rate of 95.85%. In particular, a subset of this demographic, comprising 10.82%, reported ownership of more than one residential property. For older one-child families, the average monthly consumption was 1.247 thousand yuan. Additionally, 59.14% of older one-child parents were reported to have received OISGAI or BOISUE. Most older one-child parents did not participate in social volunteer activities, at a rate of 81.41%, and the average social network strength was 2.194. With respect to family demographics, male children constituted 64.58% of the older one-child parents’ offspring, while 85.73% of all children were reported as being married. Furthermore, 57.77% of older parents from one-child families resided in an empty-nest arrangement (living alone or solely with their spouse), a proportion that was higher compared to the 42.23% who co-resided with their children. A stark contrast was observed in the distribution of one-child families between urban and rural areas, with the majority (74.65%) residing in urban areas, substantially higher than their rural counterparts (25.35%). Lastly, among these control variables, several factors demonstrated a significantly positive association with the life satisfaction of older one-child parents, including educational level, marital status, ADL, IADL, house asset, OISGAI or BOISUE receipt, participation in social activities, social network, the marital status of their child, co-residing living arrangements, and urban residence. Conversely, life satisfaction was significantly negatively associated with advanced age, the presence of chronic diseases, reliance on other sources of income, and having a female child.

### 4.2 Associations between different old-age social support and life satisfaction

The results of the REGOP model (full sample) in column (1) of Table 3 indicate that, controlling for other factors, while public financial support is positively significant only for the “Satisfied” category, the remaining three forms of social support measures show the significant positive association in both the “Satisfied” and “Very satisfied” categories of life satisfaction. In addition, most control variables, aligning with expectations, are significantly heterogeneously associated with older one-child parents’ different life satisfaction categories as well.

As for public financial support, column (1) of Table 3 demonstrates a significant positive association with the “satisfied” state (*p* < 0.01), while no significant association is found with the “very satisfied” state. As average marginal results shown in Table 7, a 1,000 RMB rise in public financial support is linked to a reduction of 1.5 percentage points in the likelihood of being “dissatisfied” and an increase of 1.7 percentage points in the likelihood of being “satisfied” on average. Conversely, the average marginal effect for the ‘very satisfied’ state is not statistically significant. With respect to family financial support, column (1) of Table 3 demonstrates a positive and significant association (at the 1% level) with the “satisfied” and “very satisfied” states. The average marginal effects in Table 7 further reveal that a 1-level increase in family financial support corresponds to a 1.2 percentage-point reduction in the likelihood of being ‘dissatisfied’ (*p* < 0.01). In contrast, it relates to increases of only 0.5 and 0.7 percentage points in the probability of being ‘satisfied’ and ‘very satisfied’, respectively (significant at the 10 and 1% levels); these magnitudes are much smaller compared to that of ‘dissatisfied’.

**Table 7:** Average marginal effects of various old-age social support on life satisfaction.

Variables	Life satisfaction		
	Dissatisfied	Satisfied	Very satisfied
Public financial support	−0.015***	0.017***	−0.001
	(0.004)	(0.004)	(0.003)
Family financial support	−0.012***	0.005*	0.007***
	(0.002)	(0.003)	(0.002)
Public time support	−0.045***	−0.027**	0.072***
	(0.014)	(0.014)	(0.012)
Family time support	−0.008***	0.001	0.007***
	(0.002)	(0.002)	(0.002)
Control variables	Control		

*p < 0.10, **p < 0.05, ***p < 0.01. Standard errors are provided in parentheses.

Regarding public time support, the results in column (1) of Table 3 show that it is positively associated with both “satisfied” and “very satisfied” states at the 1% significance level. As reported in Table 7, a 1-level increase in this variable corresponds to a 4.5- and a 2.7-percentage-point decrease in the probabilities of being ‘dissatisfied’ and ‘satisfied’, respectively. In contrast, a 7.2-percentage-point increase is observed for the probability of being ‘very satisfied’ (significant at the 1% level). With respect to family time support, column (1) of Table 3 demonstrates a positive association with both “satisfied” and “very satisfied” states (*p* < 0.01). According to the average marginal effects in Table 7, 1 level increase in family time support is associated with a 0.8 percentage-point decrease in the probability of being “dissatisfied,” but no significant relationship is found with the likelihood of being “satisfied.” Conversely, a 1-level increase relates to a 0.7 percentage-point higher probability of being ‘very satisfied’ (significant at the 1% level).

**Figure 2:**
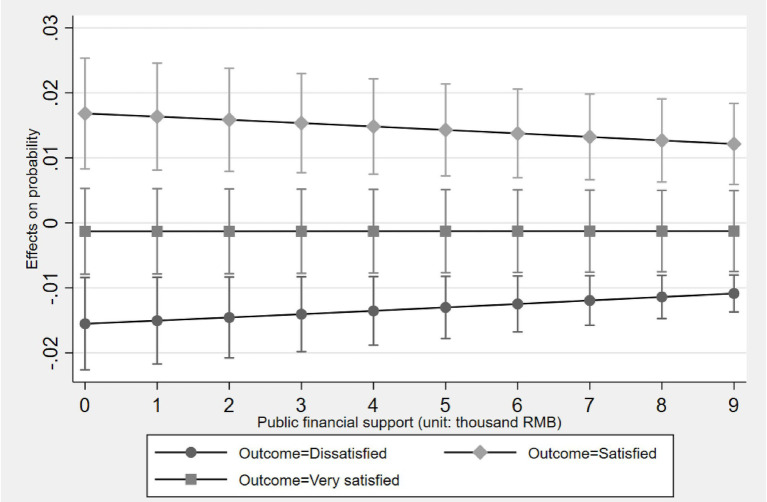
Average marginal effect of public financial support on life satisfaction with 95% ICs.

**Figure 3:**
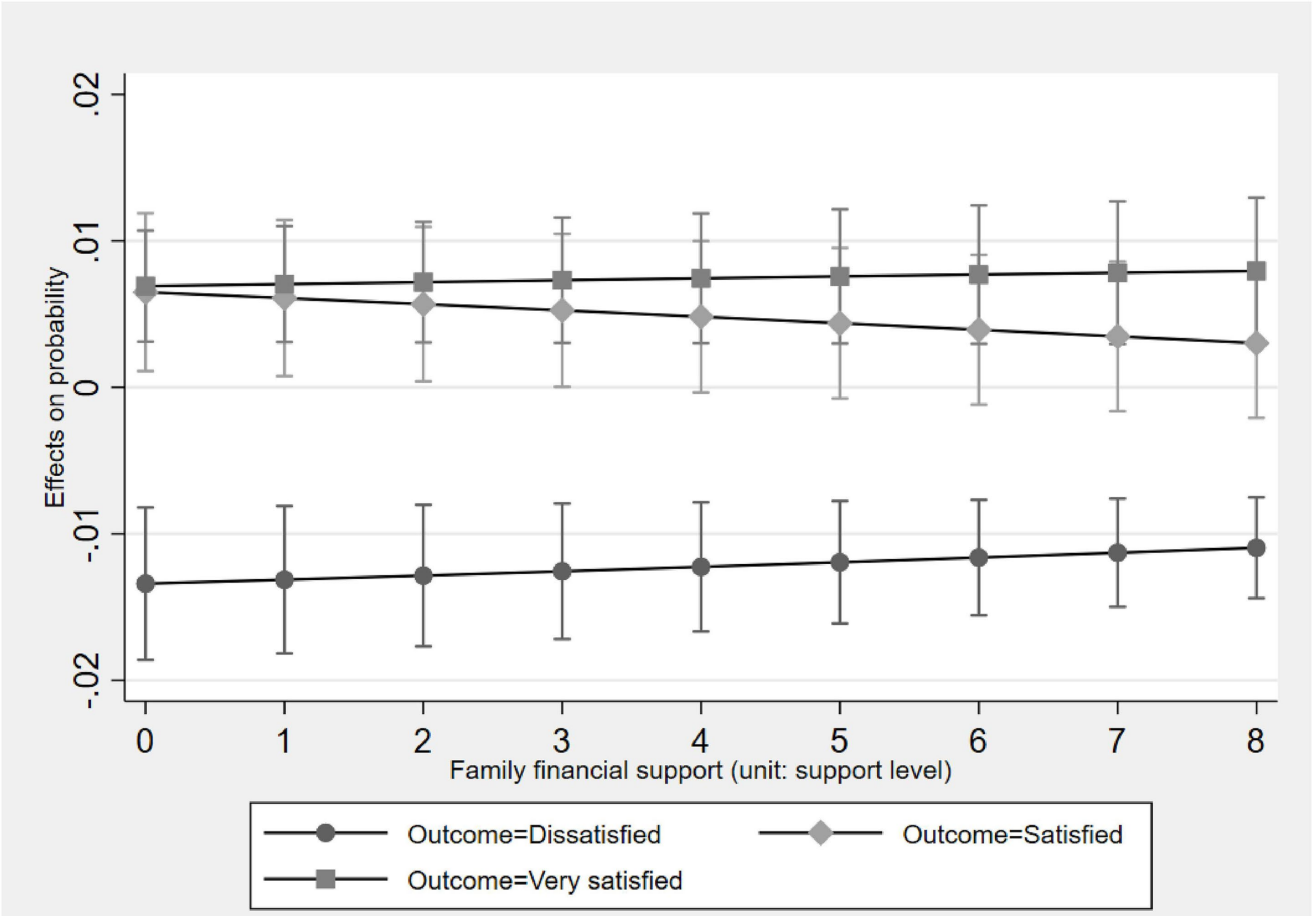
Average marginal effect of family financial support on life satisfaction with 95% ICs.

**Figure 4:**
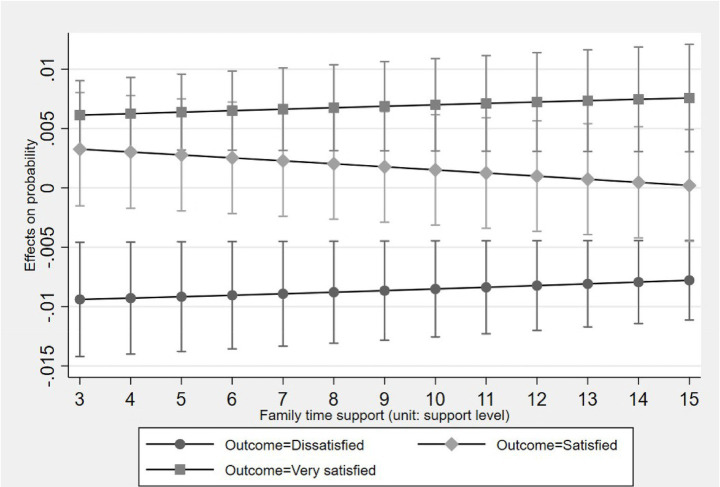
Average marginal effect of family time support on life satisfaction with 95% ICs.

**Figure 5:**
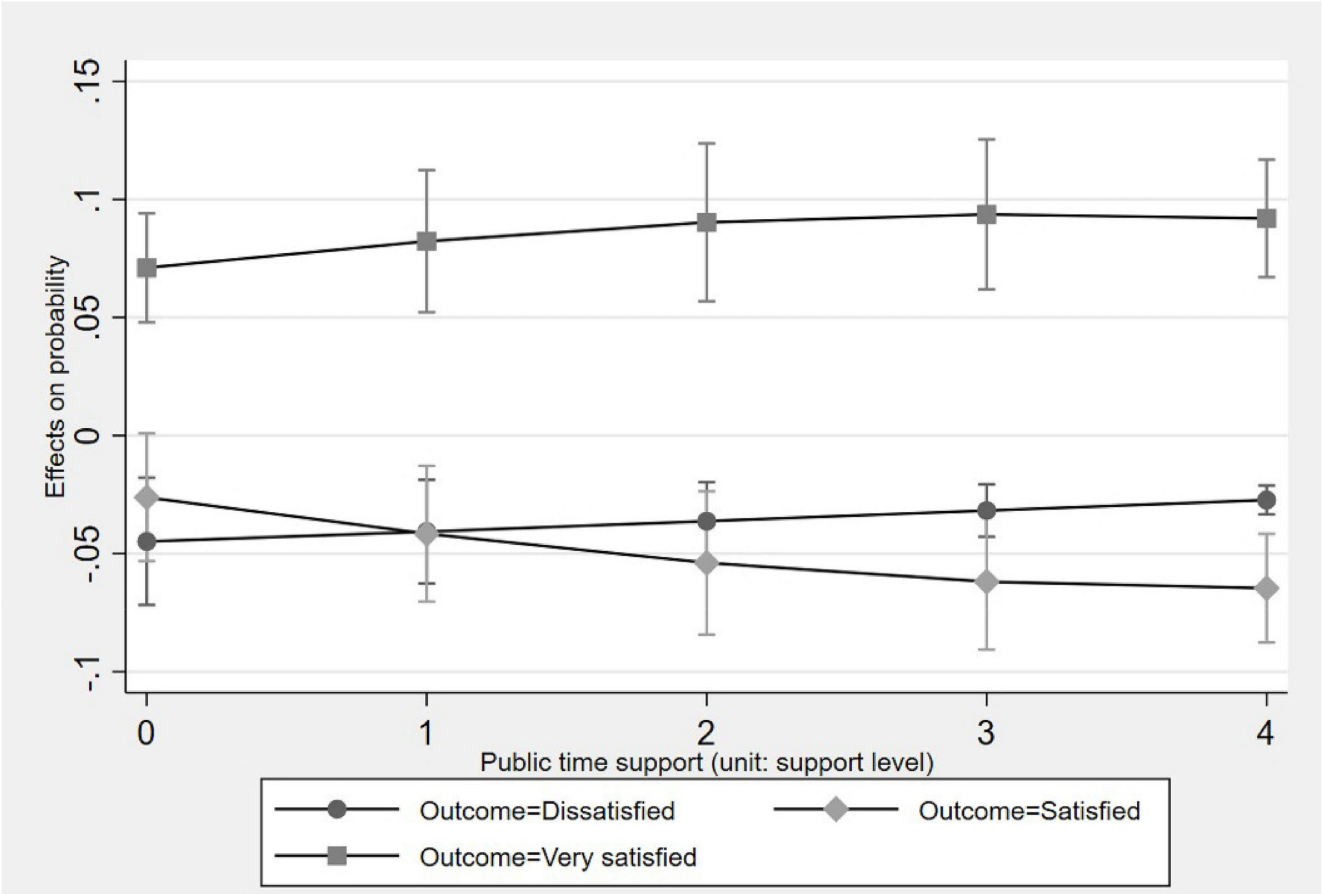
Average marginal effect of public time support on life satisfaction with 95% ICs.

Figures 2–5 display the average marginal effect plots across the entire support level range of four forms of social support on the older one-child parents’ probabilities of being in three life satisfaction categories. Taking the “public financial support” as an example, a unit change in public financial support is significantly positively associated with an increase in the probability of being in the “Satisfied” category and negatively associated with the “Dissatisfied” category across the entire public financial support range. However, for the “Very satisfied” category, the marginal effect is negative but not significant across the entire public financial support range. This result suggests that public financial support first associates with lifting older one-child parents out of “Dissatisfied” and then shifting them from “Dissatisfied” to “Satisfied,” but does not associate with further promoting them from “Satisfied” to “Very satisfied.”

Robustness checks were conducted by altering the sample size and estimation model, with results reported in columns (2) and (3) of Table 3. In column (2), the sample was narrowed to older one-child parents aged 60–70 to exclude sample selection bias; for example, individuals older than 70 might have a health or economic advantage (healthy survivor effect). In column (3), we treated life satisfaction as a continuous initial five-value variable, and a two-way fixed effects model was employed for re-estimation, controlling for individual fixed effects. As shown in columns (2) and (3) of Table 3, the results corroborated the robustness of the initial findings in column (1).

### 4.3 The relative contribution of various old-age social support to life satisfaction inequity

Using the Shapley decomposition method, the relative contribution of various old-age social support to life satisfaction inequity of older one-child parents is decomposed, with results shown in columns (1) and (2) of Table 8. The results in column (1) indicated that old-age financial support contributed 11.94% to the life satisfaction inequality, surpassing the 4.85% attributed to time support. Regarding the relative contributions of old-age family and public support to life satisfaction inequality, the results presented in column (2) of Table 8 indicate that family support accounts for 12.01% of the inequality. This figure substantially exceeds the 4.03% contribution of public support. Robustness checks using *R*^2^ decomposition based on the OLS regression model (53), presented in columns (3) and (4), confirm the main results. Column (3) demonstrates that financial support accounts for 5.70% of the inequality in life satisfaction, slightly surpassing the 4.79% contribution of time support. Likewise, the results in column (4) reveal that the relative contribution of family support stands at 6.37%, which is also higher than the 4.07% attributed to public support.

**Table 8:** Shapley value decomposition of life satisfaction inequality (*N* = 7,517).

Variables	IOP decomposition		*R*^2^ decomposition	
	(1)	(2)	(3)	(4)
	Contributions			
Financial support	11.94% (0.008)		5.70% (0.006)	
Time support	4.85% (0.003)		4.79% (0.005)	
Family support		12.01% (0.008)		6.37% (0.007)
Public support		4.03% (0.003)		4.07% (0.004)
Demographic characteristics	11.48% (0.008)	11.60% (0.008)	5.58% (0.006)	5.57% (0.006)
Physical health characteristics	17.55% (0.012)	17.56% (0.012)	44.56% (0.047)	44.53% (0.047)
Characteristics of children	3.58% (0.003)	3.68% (0.003)	0.73% (0.001)	0.75% (0.001)
Social characteristics	16.89% (0.012)	17.00% (0.012)	11.38% (0.012)	11.40% (0.012)
Economic characteristics	11.80% (0.008)	12.01% (0.008)	5.79% (0.006)	5.83% (0.006)
Time effect	7.16% (0.005)	7.16% (0.005)	10.00% (0.011)	9.97% (0.011)
Regional effect	14.74% (0.010)	14.95% (0.010)	9.48% (0.010)	9.53% (0.010)
Total	100.00% (0.069)	100.00% (0.069)	100.00% (0.105)	100.00% (0.105)

The decomposition is conducted based on pooled regression model. When conducting shapely value decomposition, four old-age social support are divided into financial support and time support based on resources content. Additionally, old-age social support is also categorized according to family and public provision channels. The values in parentheses are absolute contribution of explanatory variable to inequality of life satisfaction.

### 4.4 Heterogeneous associations between different old-age social support and life satisfaction

#### 4.4.1 Heterogeneous associations analysis related to urban and rural environment

Panel A of Table 9 presents the heterogeneous associations between social support (financial and time) and life satisfaction across rural and urban environments, revealing distinct patterns between these settings. Furthermore, the differences in coefficients between urban and rural subgroups were tested using Fisher’s test, with the results shown in columns (1), (2), and (3) of Table 10. Additionally, we corrected all unadjusted *p*-values using the Benjamini–Hochberg procedure, reporting the results as FDR *q*-values.

**Table 9:** Heterogeneity in the association between old-age social support and life satisfaction: multiple inference adjustments using Benjamini–Hochberg procedure.

Variables	(1)	(2)	(3)	(4)	(5)	(6)
	Effect size	Unadjusted *p*-value	FDR *q-*value	Effect size	Unadjusted *p-*value	FDR *q-*value
Panel A	Urban subgroup			Rural subgroup		
Public financial support	0.0907	0.000	0.000	0.0128	0.765	0.765
Family financial support	0.0628	0.000	0.000	0.0352	0.326	0.348
Public time support	0.0963	0.278	0.318	0.4950	0.073	0.117
Family time support	0.0481	0.000	0.000	0.0363	0.149	0.183
Control variable	Control			Control		
Sample size	5,588			1929		
Panel B	Solitary subgroup			Co-residing subgroup		
Public financial support	0.0658	0.038	0.076	0.1040	0.002	0.005
Family financial support	0.0520	0.017	0.039	0.0771	0.000	0.000
Public time support	0.2580	0.061	0.108	0.2590	0.091	0.132
Family time support	0.0648	0.001	0.003	0.0318	0.103	0.137
Control variable	Control			Control		
Sample size	4,681			2,836		

In different subgroups, control variables are all controlled.

**Table 10:** Fisher’s test for differences of coefficients between subgroups: multiple comparison inference adjustments using Benjamini–Hochberg procedure.

Variables	(1)	(2)	(3)	(4)	(5)	(6)
	Urban–rural differences	Unadjusted *p-value*	FDR *q-value*	Living mode differences	Unadjusted *p-value*	FDR *q-value*
Public financial support	−0.078	0.020	0.080	0.039	0.200	0.320
Family financial support	−0.028	0.350	0.450	0.025	0.040	0.107
Public time support	0.398	0.010	0.080	0.001	0.430	0.450
Family time support	−0.012	0.450	0.450	−0.033	0.180	0.320

Fisher combined tests 100 Bootstrap. H0: no difference in parameters between the two subsamples; H1: there is a significant difference between the parameters of two subsamples.

The FDR *q*-values in columns (3) and (6) of Panel A confirm that the heterogeneous associations of public and family financial support with life satisfaction across rural and urban environments remain significant following correction for multiple subgroup comparisons. Specifically, columns (2) and (5) of Panel A reveal a significantly positive association between financial support and older one-child parents’ life satisfaction in urban areas (unadjusted *p* < 0.01); conversely, no significant associations are observed in rural areas (unadjusted *p* = 0.765 and 0.326). Consistent with these findings, the FDR *q*-values reported in columns (3) and (6) are 0.000 for urban areas (far below the 5% threshold) and 0.765 and 0.348 for rural areas (far exceeding the threshold), respectively.

With respect to public and family time support, FDR *q*-values in columns (3) and (6) of Panel A suggest that only the heterogeneous association of family time support with life satisfaction remains significant after adjusting for multiple comparisons. Specifically, as shown in columns (2) and (5) of Panel A, family time support is significantly associated with life satisfaction in urban areas (unadjusted *p* < 0.01) but not in rural areas (unadjusted *p* = 0.149). This significance is confirmed by the FDR *q*-values in columns (3) and (6), which are 0.000 for urban areas (below the 5% threshold) and 0.183 for rural areas (above the threshold).

In contrast, the heterogeneous association for public time support does not survive FDR correction. Although columns (2) and (5) of Panel A show a marginally significant positive link in rural areas at the 10% level (unadjusted *p* < 0.073), with no significant effect in urban areas (unadjusted *p* = 0.278), the FDR correction reported in columns (3) and (6) renders these results insignificant. The *q*-values for both rural (0.117) and urban (0.318) areas exceed the 10% threshold.

#### 4.4.2 Heterogeneous associations analysis related to living arrangement

Panel B of Table 9 presents the heterogeneous associations between social support (financial and time) and life satisfaction, derived from subgroup regressions across different living arrangements. These results reveal distinct patterns among the different settings. Furthermore, the differences in coefficients between living arrangement subgroups were tested using Fisher’s test, with the results presented in columns (4), (5), and (6) of Table 10. Additionally, we corrected all unadjusted *p*-values for each regression and test using the Benjamini–Hochberg procedure, with the adjusted results reported as FDR q-values.

Although financial support is positively linked to life satisfaction among older one-child parents across various living arrangements, FDR-corrected results indicate insignificant heterogeneity. As shown in Panel B (columns 2 and 5), both solitary and co-residing subgroups display significant positive associations (*p* < 0.05). After FDR correction (columns 3 and 6), most associations remain significant at the 5% level, though public financial support drops to the 10% significance level (*q* = 0.076). However, Fisher’s tests (column 6 of Table 10) show no significant differences between subgroups for either public (*q* = 0.320) or family financial support (*q* = 0.107), even at the 10% level. Thus, the positive associations of financial support with life satisfaction are consistent across different living arrangements.

With respect to family and public time support, results based on FDR *q*-values reveal that only family time support demonstrates significant heterogeneity across different living arrangements following adjustment for multiple comparisons. As indicated in columns (2) and (5) of Panel B, family time support is significantly associated with life satisfaction among the solitary subgroup (unadjusted *p* < 0.01) but not among the co-residing subgroup (unadjusted *p* = 0.103). This difference persists after FDR correction: columns (3) and (6) of Panel B show a *q*-value of 0.003 for the solitary group (well under the 5% threshold) compared to 0.137 for the co-residing group (above the threshold).

In contrast, the associations for public time support do not survive FDR correction. Although both solitary and co-residing subgroups show marginally significant positive links at the 10% level in the unadjusted results (*p* = 0.061 and 0.073, respectively), the FDR *q*-values reported in columns (3) and (6) of Panel B are 0.108 and 0.132, respectively. Both values exceed the 10% threshold, rendering the associations insignificant.

## 5 Discussion

From the perspective of improving subjective well-being, this study offered empirical insights into social support policy alternatives for older one-child parents in China. First, based on significant associations of various social supports with the life satisfaction of older one-child parents, our findings suggest that the relative contribution of old-age financial support to inequality in life satisfaction is larger than that of time support. In other words, financial support, rather than time support, appears to be a more important associated factor contributing to subjective well-being for older one-child parents, highlighting a potential direction for policy prioritization. This finding stands in contrast to a qualitative study of older Chinese in the U.S. (57) and a recent quantitative study focusing on older adult patients with chronic diseases in China (24), both of which concluded that time support, such as emotional and spiritual companionship, was valued more highly than material assistance. This divergence may stem from differences in the economic and health status among the participants. On the one hand, unlike their U.S. counterparts, older adults in China face a less developed social security system and are influenced by the family planning policy, which may lead to constrained upward intergenerational financial transfers. Consequently, financial security appears to be a particularly urgent concern for older one-child parents in China at present. On the other hand, for older adult patients with chronic diseases, time support, such as healthcare assistance, remains an undoubtedly fundamental practical need for maintaining subjective well-being. Notably, this result echoes recent research on general older adults (25), despite that study focusing exclusively on family social support.

Second, our results suggest that family support accounts for a larger proportion of the inequality in life satisfaction compared to public support, which is consistent with one previous study holding that the effect of informal social support on life satisfaction is greater than that of formal social support for general older adults in China (56). This implies that social support from family members, rather than public sources, appears to be a more important associated factor contributing to subjective well-being for older one-child parents. The observation aligns with the ‘differential mode of association’ observed in caregiving, where the network of caregivers typically spans from immediate family members to social workers (58). Furthermore, our findings confirm the Confucian-influenced policy orientation observed in East Asian countries like Singapore, which prioritizes family function (59). Regarding policy improvement for one-child families, this suggests that policies aimed at reinforcing traditional family support may be a potential direction for enhancing the subjective well-being of older one-child parents compared to public support initiatives, such as community-based older adults care assistance.

Third, the positive association between old-age social support and subjective well-being varies across environments, particularly between urban and rural settings. A significantly positive association between financial support (from both public and family sources) and subjective well-being was observed only in urban areas. This may be explained by the fact that, despite a high SIOPURR reception rate in rural samples (53.36%), the median pension (90 RMB/month) was only slightly above the minimum standard. This low benefit level may lead to a diminished marginal effect that is insufficient to significantly improve the subjective well-being of older one-child parents (38). In contrast, while the reception rate in the urban sample was lower (14.71%), the median pension was substantially higher (1,353 RMB/month). Similarly, the level of family financial support was significantly lower in the rural sample (level 3: 200–499 RMB in the past year) than in the urban sample (level 4: 500–999 RMB in the past year). This disparity in financial support levels likely accounts for the observed heterogeneity in associations.

Meanwhile, family time support was only positively associated with older one-child parents’ subjective well-being in urban areas, while the positive association was not significant in rural areas. This urban–rural disparity might stem from divergent welfare environments and the older one-child parents’ misalignment of old-age expectations. In cities, robust social security covers basic needs, rendering the one-child’s high-quality time support a crucial “value-added” factor for well-being (25). Conversely, in rural areas, where low household incomes commonly drive adult single-child migration for work and pension benefits for rural ones are also insufficient, most older adults prioritize material security. Consequently, adult children’s time support might be interpreted by parents as a lack of labor market engagement or limited earning capacity. This expectation misalignment prevents time support from having a significant positive association with life satisfaction. The result of time support in the urban subgroup is consistent with research conducted in developed countries, such as the US and Japan (21, 23), which have established robust old-age support systems. However, the result of time support in the rural subgroup is inconsistent with some studies focusing on general older adults in rural China (25). The reason might be that the older adults in these studies have multiple children, implying that siblings can shoulder distinct responsibilities for old-age support, such as one providing financial aid while another offers daily care (60). This also highlights the divergence in old-age support patterns between single-child and multiple-child families.

The findings on the heterogeneous positive association of time support in different living arrangements are also interesting. Specifically, the time support from family channels is found to be associated only with the subjective well-being of older one-child parents who are living in solitary mode. We can explain this by the transformation of family responsibility in current China. As some studies have revealed, the current cohabitation motivation is driven mainly by the younger generation’s demand for “intergenerational care” (61), leading to a family ethic shift from “the old first” to “the young first,” especially for one-child families (62). This shift imposes excessive physical and psychological burdens on older adults, hindering the effectiveness of old-age care within the family (63). The older one-child parents living in co-residing arrangements receive their family time support at the expense of upward time support, such as doing housework or taking care of grandchildren for adult children, which offsets the positive impact of time support from family members. Therefore, the association between family time support and subjective well-being in different living modes is heterogeneous.

Notably, after FDR correction, public time support showed no significant association with the subjective well-being of older one-child parents in any subgroup, irrespective of rural–urban environment or living arrangement. Conversely, the REGOP model in Table 3 reveals a significant association between public time support and subjective well-being specifically for the corresponding category of ‘Satisfied’. This apparent inconsistency between the overall and subgroup analyses might seem counterintuitive. It may be attributed to sample size limitations statistically (64), the pooled analysis benefits from a larger sample size and reduced standard error, thereby achieving significance where individual subgroups do not. In fact, results in columns (2) and (5) of Table 9 still indicate significant heterogeneity in the association of public time support across subgroups based on unadjusted *p*-values. Consequently, robust evidence of such heterogeneity might be more clearly demonstrated if there were an adequate sample size.

Given that the identified associations cannot be taken as evidence of strict causality, the social policy implications discussed below should be interpreted as tentative suggestions rather than definitive recommendations. Unlike in developed countries with a well-established social security institutional system, social support policy alternatives for older one-child parents in developing countries, such as China, could consider potential prioritization of financial or material resource support, especially for one-child families in rural areas, rather than time support such as old-age services when facing the dual challenges of low fertility and aging. In East Asian contexts characterized by Confucian values, family-based support mechanisms might be a better choice. This could involve implementing financial subsidies for caregiving or establishing statutory leave for adult children from one-child families. In addition, to tailor policies to specific needs, support policy could also consider urban–rural environments and family living arrangements. Considering the urban–rural disparity, for rural older parents with only one child, boosting public pension levels and offering caregiving subsidies to their children may be preferable to simply raising enrollment rates. To address the specific needs of co-residing one-child families, policymakers could consider implementing time-support measures like community emotional counseling and community childcare assistance, thereby alleviating the extra childcare burdens borne by grandparents.

Nevertheless, the feasibility of these policy proposals still requires careful evaluation. First, regarding coordination of policies, the economic support measures for one-child families could be integrated into the existing unified urban–rural pension insurance system, significantly reducing implementation costs. Furthermore, the identification of target households is facilitated by using historical “One-Child Certificates” as a reliable verification basis. Second, to address fiscal and resource constraints, a phased and regionally stratified approach is advisable. For instance, pilot programs like the “older parents care subsidy for one-child families” have already been launched in some provinces. This step-by-step strategy allows for effective resource management and sustainable policy expansion.

Despite the insights gained from this study, the conclusions of this research are subject to certain limitations: First, owing to data constraints, we relied on unbalanced panel data, which limits causal inference and prevents an analysis of how old-age social support policies affect the subjective well-being of older one-child parents over time. Second, due to data availability, we examine old-age support with a focus only on financial support from children and SIOPURR, which might omit other material resources like property and rental income. Third, because of the lag in old-age support practices, we can only proxy public time support with some community older adults care services, which might skew subjective well-being estimation. Fourth, despite the corrections applied, the large number of subgroup tests inherently carries a risk of Type I errors; the results of the heterogeneity analysis should be interpreted as exploratory and hypothesis-generating and should be validated by future studies with larger sample sizes. Lastly, although we used the REGOP model to control for individual heterogeneity, endogeneity and reverse causality, to some extent, remain concerns. Since identifying valid instrumental variables for social support is challenging in this study, further research could exploit exogenous policy reforms or regional variations in social support provisions as potential instruments or conduct a Discrete Choice Experiment (DCE) to obtain more credible causal estimates. In the future, studies with more old-age support practices and reasonable variable measurements could also help provide more robust evidence regarding the relationship between variables.

## 6 Conclusion

For older one-child parents experiencing declines in subjective well-being, our study indicates that financial support accounts for a larger proportion of inequality in life satisfaction compared to time support, while family support appears to play a more prominent role than public support. Furthermore, regarding the rural–urban environment and living arrangements, we find that the associations of old-age social support through different channels with subjective well-being are heterogeneous. These findings contribute to the ongoing debate regarding the prioritization of resource support and family function, providing potential insights for informing social support policy alternatives for older one-child parents. Our findings also suggest that heterogeneous policy designs—specifically those that account for urban–rural disparities and living arrangements—warrant further consideration. However, given the specific context of our study, the generalization of these policy implications to regions with different socio-economic structures should still be made with caution.

## Data Availability

Publicly available datasets were analyzed in this study. This data can be found at: http://class.ruc.edu.cn.

## Appendix

**Table A1:** Proposals and pilot programs related to government intervention in support for older one-child parents.

Category		Content	Sources
Old-age financial support	Public channel	Targeting at older one-child parents, increasing their pension directly	2022 “Two Sessions” Report: Chen Xueping representative: proposing 10–20% up of pension for older one-child parents
	Family channel	Targeting at single children, providing subsidy for lost work hours associated with older adults care services	Scholars suggested local governments to offer subsidy to single children for lost work hours associated with older adults care services (28)
Old-age time support	Public channel	Targeting at older one-child parents, providing community older adults care services purchased by government	The 2014 National Health and Family Planning Commission document: the notice on pilot program on older adults care services for families having conducting family planning policy
	Family channel	Targeting at single children, providing paid nursing leave	Since 2016, regulations introduced in Henan, Fujian, Guangdong and other provinces: single child paid nursing leave

The sources are from relevant literature and news.
